# Health risk behaviours amongst school adolescents: protocol for a mixed methods study

**DOI:** 10.1186/s12889-016-3873-4

**Published:** 2016-11-29

**Authors:** Youness El Achhab, Abdelghaffar El Ammari, Hicham El Kazdouh, Adil Najdi, Mohamed Berraho, Nabil Tachfouti, Driss Lamri, Samira El Fakir, Chakib Nejjari

**Affiliations:** 1 Laboratory of Epidemiology, Clinical Research and Community Health, Faculty of Medicine and Pharmacy of Fez, B.P 1893, Km 2.2 Route Sidi Harazem, Fez, 30000 Morocco; 2 Regional Centre for Careers Education and Training of Taza, Taza, Morocco; 3 Mohammed VI University for Health Sciences, Casablanca, Morocco

**Keywords:** Health-risk behaviours, Quantitative and qualitative methods, Health education, Adolescents, School, Morocco

## Abstract

**Background:**

Determining risky behaviours of adolescents provides valuable information for designing appropriate intervention programmes for advancing adolescent’s health. However, these behaviours are not fully addressed by researchers in a comprehensive approach. We report the protocol of a mixed methods study designed to investigate the health risk behaviours of Moroccan adolescents with the goal of identifying suitable strategies to address their health concerns.

**Methods:**

We used a sequential two-phase explanatory mixed method study design. The approach begins with the collection of quantitative data, followed by the collection of qualitative data to explain and enrich the quantitative findings. In the first phase, the global school-based student health survey (GSHS) was administered to 800 students who were between 14 and 19 years of age. The second phase engaged adolescents, parents and teachers in focus groups and assessed education documents to explore the level of coverage of health education in the programme learnt in the middle school. To obtain opinions about strategies to reduce Moroccan adolescents’ health risk behaviours, a nominal group technique will be used.

**Discussion:**

The findings of this mixed methods sequential explanatory study provide insights into the risk behaviours that need to be considered if intervention programmes and preventive strategies are to be designed to promote adolescent’s health in the Moroccan school.

## Background

Adolescence is a critical transitional period that includes the biological changes of puberty, the need to increase independence, preoccupation with the self, and normative experimentation [1, 2]. During the transition from childhood to adulthood, adolescents struggle to make lifestyle choices and establish patterns of behaviour that affect both their current and future health.

A number of health risk behaviours begin in adolescence that affect health both at the time and in later years. Some of these behaviours contribute to the leading causes of mortality and morbidity among adolescents, such as suicide attempts, injuries and the various risks associated with unprotected sexual behaviour, conditions related to tobacco or alcohol use and overweight or obesity [3–5]. The majority of adolescent death and illness are caused by risk behaviours that can be grouped into four categories: tobacco, alcohol and drug use; dietary behaviours; physical activity; and sexual behaviours [6, 7]. These key health-risk behaviours are often the focus of prevention strategies for non-communicable diseases and some sexual conditions [8, 9].

Behaviour change is based on social cognitive theory and social-ecological model of health, which emphasizes a dynamic interaction among cognitive, behavioural, and environmental factors over the life course of individuals, families, and communities contributing to the health of populations [10]. An understanding of risk and protective factors at multiple levels, including the adolescent, family, school, and community, has influenced intervention development [4]. At school, a multi-behaviour approach is the most effective way to promote healthy behaviours among adolescents [5, 6].

According to Centers for Disease Control and Prevention (CDC), The Global School-based Student Health Survey (GSHS) in 2006 and 2010 demonstrated that most health-risk behaviours among adolescents in Morocco are tended to increase [11]. Recently, in the Arab Teens Lifestyle Study (ATLS) survey, Hamrani and collaborators found a high prevalence of sedentary behaviours and physical inactivity among Moroccan adolescents in Kenitra city public schools with 45% of Moroccan participants reporting television viewing for more than 2 h per day and 38% engaged in computer use for a similar period [12]. Most adolescents also reported unhealthy dietary habits including skipping breakfast, little consumption of fruits and vegetables and approximately 50% of adolescents consumed sugary drinks more than three times per week [12]. In addition, in a recent study on psychoactive substance use in the North Central region of Morocco, the prevalence of smoking in adolescents was estimated as 16.1% and cannabis use recorded the highest lifetime prevalence of 8.1%, followed by alcohol 4.3% [13].

Health education is an integral part of the overall education of students. In many educational systems, health education constitutes an independent discipline. However in other systems, like that in Morocco, health goals are implemented in the curricula of several disciplines. The focus of health education is to teach the skills that enable students to make healthy choices and avoid health-risk behaviours [14]. Poor health related behaviours, as well as malnutrition and limited knowledge about nutrition have been reported in studies conducted among Moroccan adolescents [12, 15, 16]. A study conducted in Morocco, evaluating the content of school textbooks in relation to health lessons, concluded that the lessons in these textbooks are inadequate, inaccurate, or out of date, and hence need improvement [17]. In Morocco, the curriculum was also primarily based on the biomedical model and included a few instructions issued from the social health model [18]. A high quality health education programme is the cornerstone of promoting healthy behaviours in the school setting.

The World Health Organization (WHO) [19] and recently the commission on adolescent health and wellbeing [20] emphasize that national level data are necessary to inform national decision makers in relation to the most relevant policies to support adolescents. Additionally, although many countries had useful data, the scope of differences at political, socio-cultural and religious levels, together with different availability of resources, suggests that each country prerequisites its own data in order to better target unhealthy behaviours of adolescents. In Morocco, most studies of adolescents’ health-risky behaviours have been conducted with a quantitative approach. Limited qualitative data are available regarding adolescent behaviour.

### The study aims

The aim of this mixed methods study is to investigate the health risk behaviours of Moroccan adolescents aged 14 to 19 years with the goal of identifying suitable strategies to address their health concerns. The opinion of parents and teachers on adolescent’s behaviours was also explored. On the basis of the findings, interventions for promoting healthy behaviours in school will be developed. This research design manuscript sets out to describe the detailed methods used to collect data that can inform future efficacious health education interventions targeting adolescents.

## Methods

### Design

**Fig. 1:**
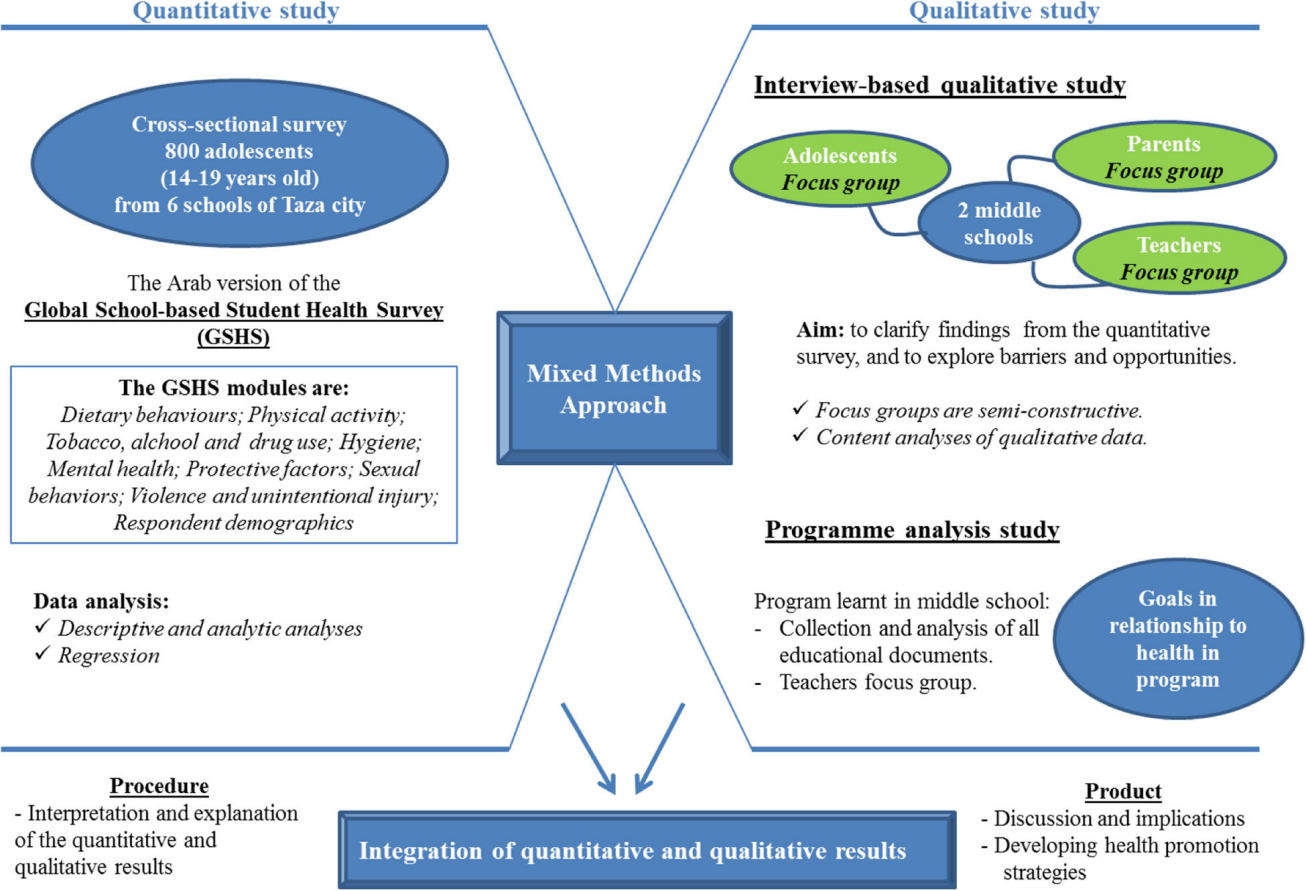
Overview of the study design

The present study utilises a mixed methods design. The explanatory progressive approach is a two-phase mixed methods design that begins with the collection of quantitative data, followed by the collection of the qualitative data to explain and enrich the quantitative findings [21]. In this study, the priority was given to the quantitative data. After analysis of the questionnaire data, qualitative data collection was conducted regarding findings that required additional explanation. In addition, a description of all goals in relationship to health in the educational programme of middle schools was conducted to determine if the existing programme meets the international standards for adolescent health behaviours. An overview of the study design is presented in the Fig. 1.

### Phase I: quantitative study

We designed a school-based cross-sectional study to investigate health risk behaviours and their correlates in a representative sample of adolescents aged between 14 and 19 years old in Taza city.

#### Sample size and sampling method

The sample size was estimated using the Epi-info software based on a population of 30,000 students (http://www.openepi.com/). The sample size (*n* = 380) was calculated based on the 95% confidence level and the population proportion has been assumed to be 0.50, as this magnitude yield the maximum possible sample size required. A multistage stratified cluster random sampling method was used to select the sample, and a design effect coefficient of two was chosen resulting in a final sample size of 760 (380 × 2).

The sampling frame was the roster of middle and secondary schools for the whole Taza city (17 middle and secondary schools). Stratified cluster random sampling was used to produce more precision and better representatives of the study population. In the first stage, two schools were randomly selected with each three neighbourhoods defined by socio-economic level (disadvantaged, average, and advantaged). At the second stage, four classes were randomly selected in each selected school on the day of the survey. All students in the selected classes were invited to participate in the study. Because of differences in class size between schools, the sample sizes for the participating schools differed.

#### Data collection

The Arab version of GSHS was administered to all participants, together with a questionnaire that included socio-demographic questions. Details and data of the GSHS can be accessed at http://www.who.int/chp/gshs/methodology/en/. The GSHS 10-core questionnaire modules address the leading causes of morbidity and mortality among children and adults worldwide: tobacco, alcohol, and other drug use; dietary behaviours; hygiene; mental health; physical activity; sexual behaviours that contribute to HIV infection, other sexually transmitted infections; unintentional injuries and violence and protective factors.

#### Data analysis

Quantitative data were coded, entered, cleaned for completeness and inconsistencies, and analysed using SPSS version 19 (SPSS, Inc, Chicago, IL, USA) at the faculty of Medicine and Pharmacy of Fez. Descriptive statistics will be presented as means ± standard deviations (SD) or proportions. Bivariate logistic regression analyses were used to assess associated factors. The level of significance will be set at *p* < 0.05.

### Phase II: qualitative study

A qualitative method was designed to provide deep and broad understanding adolescent behaviours. This phase was included in interviewer-administered qualitative survey and a programme analysis. Parents, teachers and adolescents were invited to participate in the interview study.

### Interview-based qualitative study

#### Sample size and sampling method

Adolescents were recruited from two middle schools (disadvantage and advantage according to socio-economic level) that participated in the quantitative study. From each school adolescents from the last year in middle school between the ages of 14-16 years and their parents were interviewed. This age group of students was selected because they study in their educational programs all the items related to health risk behaviours. Teachers of disciplines that are concerned to health risk behaviours were randomly selected from selected schools (with respect of age and sex) to participate in this study. Rather, sampling continues until data saturation is reached. It is expected that gathering information from different sources will offer a high-quality qualitative study which will provide a more complete view of adolescents’ health risk behaviours.

#### Data collection

Data were collected through focus groups (FGs) rather than using individual interviews. FGs discussions are considered the most attractive and efficient investigation methods [22]. Each group was homogeneous concerning the experience as well as the scholarly and the family environment which they come from. The participants were selected in order to obtain a wide array of different opinions. In each focus group there were 4 to 8 participants. In the FG of adolescents and their parents, the aim was to clarify findings from the quantitative survey, to explore barriers and opportunities of behaviours and to identify their suggestions for intervention. The topics covered in the FG are about physical activity, dietary habits, tobacco, alcohol and drug use, and sexual behaviours. Teachers’ FG was designed to explore barriers and opportunities to adopt healthy behaviours by adolescents in relationship with the educational programme. Suggestions on intervention are also explored with teachers. The semi-structured FG interviews continued until data saturation were achieved, that is, no new themes emerged. The FG interviews were audio-recorded and transcribed verbatim.

#### Data analysis

The qualitative data were analysed using a conventional content analysis approach. This method allows gathering data directly from participants without imposing pre-conceived categories and previous theoretical perspectives. Rather, knowledge generated from content analysis is based on the unique views of the participants and is rooted in the text data [23].

Each audio-taped interview was accurately transcribed and the text was coded and codes were rephrased into a shorter code phrase in the second phase of the analysis. Next, those phrases with similar meanings were brought together and subcategories generated. Finally, themes emerged through further abstraction [24, 25]. A sociologist experienced in qualitative research will lead all these steps.

### Programme analysis study

This phase was designed to describe the content and goals of the middle school health educational programmes. In the Moroccan education system there are five levels: early years, primary, middle, secondary and higher education. School attendance is compulsory up to the age of 13 years. The secondary cycle of school studies in Morocco is three years in duration and is open to students who have successfully completed nine years of basic education.

#### Data collection

Educational documents such as programme and pedagogic instructions and other references in relationship with the programme developed for middle school by the ministry of education were collected. FGs with teachers about the programme in middle schools were also explored in this phase. The topics covered in the FG are about the formal and hidden curriculum in term of health education. The interviews were audio-recorded and transcribed verbatim.

#### Data analysis

Content analysis will be conducted as described previously in the interview-based qualitative study. In brief, a description of health goals in all disciplines learnt in middle school followed by extraction of its characteristics (items, objectives, time devoted to each goal).

### Ethical considerations

Informed consent was obtained from participating teachers and parents of students, and an approval was obtained from students for conducting the survey. This study was approved by the Faculty of Medicine and Pharmacy of Casablanca Research Ethics Committee and the National Control Commission for the Protection of Personal Data (A-RS-193-2015).

## Discussion

The integration of quantitative and qualitative findings in this study will result to a more comprehensive picture on adolescent health risk behaviours. The combination of both quantitative and qualitative methods enables a deep analysis of the findings [21]. The results may contribute in identifying factors that should be targeted in future interventions for promoting healthier behaviours in adolescents. To obtain opinions about strategies to reduce Moroccan adolescents’ health risk behaviours, a nominal group technique (NGT) will be used [26].

Unhealthy adolescent behaviours can become long-term risk factors for chronic health conditions in adulthood [27]. The health and well-being of adolescents is influenced largely by the world in which they grow and the people that surround them. Therefore, producing healthy school environments can have numerous benefits in improving health, well-being, and academic achievement [28]. Also, adolescents who are educated and healthy are more likely to become contributing members of society and contributors to economic success. As a consequence, it is expected that findings from this strategic research endeavour will be substantial and very beneficial from public health perspectives. Additionally, results from this research may be recognize the need for programmes that teach children and teenagers the basic concepts of health and fitness and how to improve present and future health status [14, 28].

The main strength of this research project is the use of mixed methods that enables a more complete picture on adolescents’ health-risk behaviours, and hence developing effective interventions [21, 29, 30]. Potential limitation may be under-reporting many themes that involve sensitive health concerns. This would be related to the extent of socio-cultural and religious concerns.

In conclusion, this research provide insights into the risk behaviours that need to be considered if intervention programmes and preventive strategies are to be designed to promote adolescent’s health in the Moroccan school.

## Abbreviations

ATLS: Arab Teens Lifestyle Study
CDC: Centers for Disease Control and Prevention
FGs: Focus groups
GSHS: The Global School-based Student Health Survey
NGT: Nominal group technique
SD: Standard deviations
WHO: World Health Organization
